# Allosteric Modulation of G Protein Coupled Receptors by Cytoplasmic, Transmembrane and Extracellular Ligands

**DOI:** 10.3390/ph3103324

**Published:** 2010-10-25

**Authors:** Naveena Yanamala, Judith Klein-Seetharaman

**Affiliations:** Department of Structural Biology, School of Medicine, University of Pittsburgh, 3501 Fifth Avenue, Pittsburgh, PA 15260, USA

**Keywords:** rhodopsin, metabotropic glutamate receptors, allosteric network, communication, membrane proteins

## Abstract

G protein coupled receptors (GPCRs) bind diverse classes of ligands, and depending on the receptor, these may bind in their transmembrane or the extracellular domains, demonstrating the principal ability of GPCRs to bind ligand in either domains. Most recently, it was also observed that small molecule ligands can bind in the cytoplasmic domain, and modulate binding and response to extracellular or transmembrane ligands. Thus, all three domains in GPCRs are potential sites for allosteric ligands, and whether a ligand is allosteric or orthosteric depends on the receptor. Here, we will review the evidence supporting the presence of putative binding pockets in all three domains of GPCRs and discuss possible pathways of communication between these pockets.

## 1. Introduction

**Figure 1:**
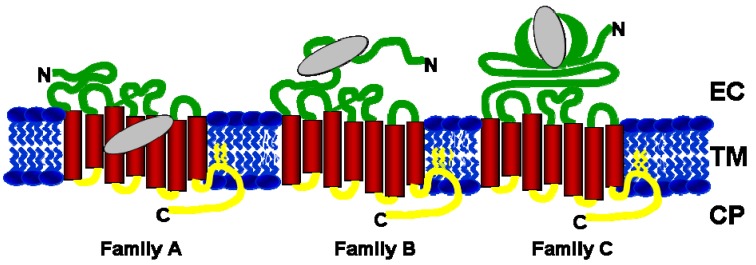
Schematic diagram of representative structural organizations of GPCRs highlighting the most common endogenous ligand binding site locations for each of the major GPCR family members. Extracellular and cytoplasmic loops are colored in green and yellow, respectively. Transmembrane helices are colored in red and represented as cylinders. The membrane bilayer is shown in blue color. The endogenous (orthosteric) ligand binding site in each family is represented by an ellipse colored in grey.

GPCRs form the largest and most diverse family of cell surface receptors. The primary function of these membrane bound receptors is to mediate the communication between the cell and its environment by responding to signals via binding and activation of intracellular G proteins, in turn, initiating cellular responses. These receptors respond to a wide variety of signals including light, ions, peptides, hormones, odorants and neurotransmitters. GPCRs are structurally characterized by seven transmembrane (TM) helices, dividing each GPCR protein into extracellular (EC), cytoplasmic (CP), and TM domains (Figure 1). For this reason, GPCRs are also known as 7TM proteins or serpentine receptors. The seven TM segments are connected by EC loops (E1, E2, and E3) and CP loops (C1, C2, and C3). The amino- (N-) and carboxy- (C-) termini of GPCRs are located at the EC and CP sides of the receptors, respectively.

The GPCR family is the most important family of receptors with more than 1,000 human sequences deposited [1,2], and more than 8,000 sequences including other organisms [3]. Many of the sequences are associated with various disease conditions and mutations in GPCR genes have been linked to diabetes [4], cancer [5,6,7], central nervous system disorders [8], obesity [9], inflammation [10], cardiac diseases [11] and others. With almost 50–60% of drugs in the market targeting GPCRs, they are the most important family of receptors in the drug discovery field [8]. Based on sequence homology and pharmacological considerations, the GPCR super-family is subdivided into six sub-families [1,12]. They are: Class A or the rhodopsin like or adrenergic receptor like family, Class B or the secretin receptor family, Class C or the metabotropic glutamate receptor like, Class D or the Fungal mating pheromone receptors, Class E or the cyclic AMP (cAMP) receptors, and Class F the frizzled or smoothened receptors. 

GPCR drug discovery is shifting away from the traditional “one ligand – one receptor – one effect” centered view to incorporate more complex schemes of multiple ligands binding to their receptor(s) with varying functional effects and mechanisms of actions. In these schemes, GPCR oligomerization and allosteric regulation have become important modulatory components [13]. Allosteric regulation in particular has raised high hopes for GPCR drug discovery because it provides a platform for novel avenues for the application of medicinal chemistry. Most of the drugs developed in the past decade are either agonists or antagonists that bind competitively to GPCRs, where “competitive” indicates the molecule binds at the same binding site as the endogenous ligand. Such drugs are often found to exert side effects. Recently, attention has therefore been given to the development of drugs that do not target the orthosteric ligand binding site, but instead, bind to other sites on receptors and modulate receptor signaling. Such types of drugs are referred to as allosteric modulators. Binding of these modulators at the allosteric site is communicated to the endogenous or orthosteric ligand binding pocket via long-range conformational changes and these, in turn, affect receptor function. The modulation of receptor function is either positive or negative, stabilizing the active or inactive conformations of the receptors, respectively. Depending on whether receptor function is enhanced or inhibited, the allosteric modulators are referred to as positive or negative allosteric modulators, respectively.

Developing allosteric modulators as opposed to orthosteric agonists and antagonists that directly target the endogenous or orthosteric ligand binding site is advantageous for several reasons. In the absence of the endogenous ligand most allosteric modulators do not exert any effect, thus preserving the physiological effects of the receptor [14,15]. Allosteric modulators only act when the endogenous ligand is bound to the receptor. Therefore, these ligands have the potential to exert fewer side effects, even when added in large excess, as compared to orthosteric ligands that directly affect signaling [14]. Further, as allosteric ligand binding sites are generally found to be located in non-conserved regions of receptors, as opposed to the highly conserved orthosteric ligand binding sites, it is possible to develop highly-selective ligands that can target subtypes of the receptors that are otherwise difficult to differentiate between [14,16]. Finally, allosteric modulators also provide a unique advantage in modulating the receptors that have peptides or small proteins as endogenous ligands. Because these endogenous ligands are relatively large and usually span an extensive area of interaction interface on the receptor, it is difficult to develop synthetic molecules that could directly target the orthosteric or endogenous ligand binding pocket in its entirety [15,17,18]. 

Thus, allosteric regulation – more so than GPCR oligomerization - has raised high hopes because it is readily amenable to medicinal chemistry to explore the novel binding pockets and potential development of ligands with better pharmacological profiles. However, optimally exploiting the promise of GPCR allostery for drug discovery will require a molecular understanding of the structural basis and molecular mechanisms of allostery in GPCRs. It is the aim of this review to discuss some recent ideas on these putative structural mechanisms and the review is structured as follows. In Section 2, we will review orthosteric and allosteric ligand binding pockets in the three major classes of GPCRs, with emphasis on their localization with respect to the three structural domains that GPCRs are composed of, the EC, TM and CP domains. In Section 3, we will use the Class A and prototypical GPCR, rhodopsin, to illustrate these three general pockets and discuss communication between them. Although rhodopsin is not a receptor for which allosteric regulation is well known, it is structurally the best characterized GPCR and can therefore serve to provide a model for the molecular nature of the pockets and the consequences of ligands binding to them for receptor structure and dynamics. The presence of a cytoplasmic ligand binding pocket in particular is relatively novel and we will review the recent evidence for this pocket in rhodopsin. In Section 4, we will then extend the structural lessons learnt from study of rhodopsin to other GPCRs where allosteric modulation is better known. In particular, we will establish an analogy between rhodopsin and metabotropic glutamate receptors (mGluRs). mGluRs are Class C GPCRs where the ligand binds to an independent folding unit in the EC domain, and many allosteric ligands have been identified which bind to the TM domain and modulate the functional responses of these receptors to activation by binding of ligands to the orthosteric, EC binding pocket. In particular, we provide the evidence supporting the hypothesis that the allosteric site of Class C GPCRs is highly similar to that of the orthosteric TM site in Class A GPCRs, and that the pockets are analogous in their function for receptor activation. In Section 5, we will speculate briefly on the putative interaction network pathway of signal communication between the three ligand pockets in GPCRs. We will conclude with a summary in Section 6.

## 2. Orthosteric and Allosteric Ligand Binding Sites in GPCRs

In Class A GPCRs, the largest and most diverse group of GPCRs, the endogenous ligand binding pocket is typically in the TM domain, near the EC domain (Figure 1, Family A). In contrast, for Class B and C the endogenous ligand pocket is almost exclusively located in the EC domain. This is also true for some Class A GPCRs, but the vast majority of members of Class A bind ligands in their TM domains. Chemokine receptors are amongst the most notable exceptions. These receptors bind to their respective endogenous chemokine ligands at the EC domain similar to Class B and C receptors (Figure 1, Family B and C). When endogenous ligands bind to GPCRs either in the EC or TM regions, the event causes conformational changes in the TM domains of the receptors, which are transmitted to the CP domain. Thus, regardless of whether the ligand binds in the EC or TM domain, its interaction with the receptors activates a switch transducing the ligand binding signal to the G proteins that bind to the receptors in their CP domain. The G protein then acts to either stimulate or inhibit the production of intracellular secondary messengers, which in turn trigger various signal transduction cascades. Even when ligands bind in the TM domain, they generally do so in the top one-third of the TM helices, and binding typically involves the EC loops and N-terminus, which frequently play a major role in ligand binding. In contrast, the opposite end of the TM domain towards the CP side and the CP loops and the C-terminus are critical for the direct interaction with downstream signaling proteins, most importantly G protein, GPCR kinases and arrestins, but for some receptors also involving many other proteins.

Recent studies have reported allosteric modulators for all major subfamilies of GPCRs (Table 1). For Class A receptors, allosteric modulators have been identified for many receptors and their subtypes including muscarinic, adenosine and adrenergic receptors. In particular, the muscarinic acetylcholine receptors are the best studied receptor type among the Class A receptors identified to be allosterically modulated [19,20]. As described above, in most members of Class A GPCRs, the endogenous ligand binding site is located in the TM domain near the EC side (Figure 1). Several structural studies have identified that the allosteric site in muscarinic acetylcholine receptors comprises EC loops 2 and 3 and part of TM helix 7 [21,22,23]. In contrast, for the chemokine receptors belonging to the same Class A family where the endogenous ligand binding site is situated in the EC side, two allosteric binding sites have been proposed. One of them is located in TM helices 1, 3, 5 and 7 [24] and the other in the CP domain [25]. In the case of family B receptors, it was shown by mutagenesis studies that the allosteric small molecule ligand binding site is composed of TM helices 3, 5 for the corticotrophin releasing factor receptor [26,27] and helices 2, 3 for glucagon-like peptide receptor [28], towards the EC side of the receptors. Finally, for family C GPCRs, in particular for mGluRs, it was shown by mutagenesis that allosteric modulators bind at the interface between the TM and EC domains similar to most of the endogenous ligand binding sites in class A GPCRs. The allosteric ligand binding site was localized to TM helices 3, 5, 6 and 7 [29,30]. It was also shown that allosteric mGluR ligands can either act as positive or negative modulators of mGluR activity in response to glutamate or glutamate analogs, enhancing or suppressing the responses, respectively [31]. Small changes in the chemical structures of ligands were shown to switch their modulatory effects. For example, 4,4’-difluorobenzaldazine (DFB-4,4`) is a negative modulator for mGluR5, while 3,3’-difluorobenzaldazine (DFB-3,3`) is a positive modulator for the same receptor [32]. For another member of this family, the calcium sensing receptor, previous studies have shown that the allosteric ligand binding site is composed of TM helices 2, 3, 6 and 7 [33,34,35]. 

**Table 1:** Members of the major subfamilies of GPCRs exhibiting allosteric modulation. The data shown in the table is summarized from a recent review [15]. The location of the orthosteric and allosteric ligand binding sites established for some of the receptors are mentioned respectively.

GPCR Family	Receptor Details	Endogenous ligand binding site	Allosteric Ligand binding site	References
Family A	Adenosine A1, A2A, A3	TM helices 3, 5, 6 and 7	EC domain (top of helices 6 & 7)	[19,20]
	Adrenoreceptors α-1, -2A, -2B, -2D	TM helices 2, 3, 5, 6 and 7		
	Chemokines CXCR1-4, CCR1,3,5	EC domain	TM and CP side	[24,25]
	Dopamine D1, D2	TM helices 2, 3, 5, 6 and 7		
	Muscarinic M1-M5	TM helices 3, 5, 6 and 7	EC and CP domains	[36,37]
	Serotonin	TM helices 3, 5, 6 and 7		
	Rhodopsin	TM helices 3, 5, 6 and 7	CP domain	[43,44,45,46]
Family B	Corticotropin Releasing Factor 1	EC domain	TM helices 3,5	[26,27]
	Glucagon	EC domain		
	Glucagon Like Peptide-1	EC domain	TM helices 2,3	[28]
Family C	Metabotropic Glutamate Receptors 1-5 and 7	EC domain	TM helices 3, 5, 6 and 7	[29,30,31,32]
	Calcium Sensing Receptor	EC domain	TM helices 3, 5, 6, and 7	[33,34,35]
	GABA-B	EC domain		

The presence of a CP allosteric binding pocket in chemokine receptors and muscarinic acetylcholine receptors (Table 1) may seem surprising given the general location of ligand binding pockets in EC and TM domains. However, the CP domain is in fact the interaction site for the conserved “ligands” for all GPCRs, the proteins of the G protein, kinase and arrestin families. While these are not small molecule ligands, the fact that they need to form an interaction surface with the receptor requires that a pocket for this interaction be present in the receptor. Not surprisingly, small peptides representing for example the G protein, also bind to the receptors – albeit with lower affinity [38,39]. The CP protein-protein interaction surface pocket may also be exploited by other ligands – proteins or small molecules, and binding may exert allosteric effects on the receptors. Thus, for example, the interaction of the CP scaffolding protein, Na^+^/H^+^ exchange regulatory factor 1 (NHERF1) with agonist bound parathyroid hormone receptor (PTH), a class B GPCR was found to regulate receptor desensitization [40]. Binding of NHERF1 at the CP domain of the PTH receptor displaced β-arrestin, inhibiting its interaction with the receptor [40]. NHERF1 binding also affected binding of EC peptide ligands to the receptor [41]. Recently, it was discovered that small organic and inorganic molecules can indeed bind to the CP domains of GPCR, reviewed in the following section with particular emphasis on the prototypical Class A GPCR, rhodopsin.

## 3. An Allosteric Ligand Binding Pocket in the Cytoplasmic Domain of Rhodopsin

Rhodopsin is the mammalian dim light photoreceptor in rod outer segments in which the ligand, 11-*cis* retinal (a vitamin A derivative), is covalently attached to the receptor via Schiff base linkage to a lysine in the TM region towards the EC domain. On light-activation, 11-*cis*-retinal isomerizes to all-*trans* retinal, resulting in a series of intermediate conformational states. Of these, the Meta II state is considered to be the active state of the protein and is an initiator of the visual signal transduction cascade. As with other GPCRs, all the proteins involved in signaling interact with rhodopsin at its CP domain. While 11-*cis-* and all-*trans*-retinal (as well as several other retinal isomers) are endogenous (or orthosteric) ligands for rhodopsin and bind in the TM region, it was recently shown that rhodopsin can also accommodate allosteric ligands in the CP domain [42,43,44,45,46].

**Figure 2:**
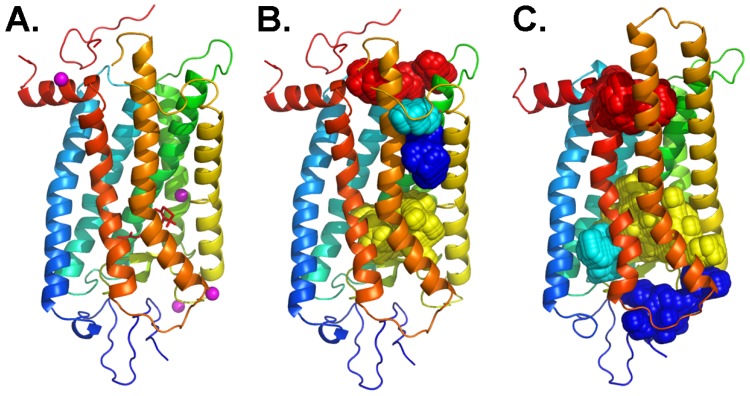
Crystal structures of rhodopsin showing orthosteric and allosteric binding sites. Cartoon representation of (A) rhodopsin (PDBID: 1L9H) with the different zinc binding sites, as well as (B) rhodopsin (PDBID: 1F88), and (C) opsin (PDBID: 3CAP) both highlighting the top 4 potential binding pockets predicted using Pocket-Finder tool implemented based on the Ligsite algorithm [47]. The top 1, 2, 3 and 4 predicted binding pockets are represented as spheres and colored in yellow, red, blue and cyan, respectively. The zinc metal ions are colored in magenta and rendered as spheres. The endogenous ligand 11-*cis* retinal is rendered as sticks and colored red in A. The N- to C-terminus of protein is colored from blue to red.

An illustration for the capacity of different ligands binding to rhodopsin is provided by an analysis of cavities in rhodopsin crystal structures using the Pocket-Finder tool [47]. The prediction of ligand binding pockets in 11-*cis*-retinal bound rhodopsin and retinal-ligand free opsin using Pocket-Finder software is shown in Figure 2B-C. Several putative ligand binding pockets were found, including the known 11-*cis*-retinal binding pocket. The top four predicted pockets for rhodopsin and opsin are shown in Figure 2B and 2C, respectively. In addition to predicting the 11-*cis*-retinal pocket with high probability (Figure 2B-C, yellow spheres), this method also resulted in another high probability site at the CP domain in both rhodopsin and opsin structures (Figure 2B-C, red spheres). This prediction supports the idea that there is a CP ligand binding pocket in rhodopsin.

Several lines of experimental evidence not only validate the prediction of an allosteric ligand binding pocket, but also demonstrate that binding of such ligands will modulate the structure and function of the proteins. Specifically, rhodopsin binds metal ions, anthocyanins and porphyrin compounds in an allosteric fashion. Rhodopsin shows altered stability when bound to any of these ligands, and its function is modulated. In particular, sensitivity to light of low intensities and of longer wavelengths is altered in the presence of porphyrins [48,49]. Specifically, binding of the anthocyanin compound, cyanidin-3-glucoside (C3G), a phenolic naturally occurring anthocyanin compound, enhanced the rate of reaction of opsin with 11-*cis*-retinal, such that regeneration in rod outer segment preparations was increased [49]. Using molecular modeling studies and NMR spectroscopy, it was demonstrated that C3G binds at the CP side of rhodopsin [43,44]. The regeneration of rhodopsin from opsin and 11-*cis*-retinal was enhanced upon binding of C3G [43] and activation of the G protein transducin was slightly inhibited [43,44]. Thus, this study provided a molecular mechanism for the previous observation that C3G accelerates rhodopsin regeneration by two-folds [49]: modulation of rhodopsin structure via long-range mediated structural effects enhances access to the retinal binding pocket. This conforms to the classical definition of allostery applied to rhodopsin: the binding of a small molecule ligand to the CP pocket alters the binding of the orthosteric retinal ligand.

The chlorophyll derivative Ce6 has been proposed to enhance sensitivity of rhodopsin to red light in deep-sea fish rhodopsin [50,51]. Indeed, *in vivo* studies with salamander and mouse models have confirmed that Ce6 can effectively enhance vision in other animals [52]. A decrease in the 500 nm peak of a UV-Visible spectrum of salamander rhodopsin was observed by illuminating the sample at 668 nm, a wavelength at which Ce6 absorbs while rhodopsin essentially does not [51]. Additionally, mice administered with Ce6, showed an two fold increase in electroretinogram b-wave amplitudes (the electrical signaling to brain upon a flash) as a response to red and blue light [52]. Ce6 in the presence of the divalent metal ion, Zinc (Zn^2+^), showed remarkable stabilization of the secondary structure of rhodopsin to thermal denaturation [42]. 

Independent of Ce6, Zn^2+^ was also shown to modulate the effects of orthosteric ligands in many GPCRs alone [53,54,55,56], including rhodopsin [57]. Two different Zn^2+ ^binding sites were proposed for rhodopsin, (1) a high affinity site – in the TM domain, and (2) a low affinity site – closer to the EC domain (Figure 2A). While, Zn^2+ ^binding at the TM domain stabilized the interaction between 11-*cis*-retinal and rhodopsin, its binding at the EC domain caused destabilization [42,58,59]. Recently, it was proposed that addition of Zn^2+ ^shown to act as an allosteric modulator of rhodopsin, either by stabilizing its structure or destabilizing its interaction with the orthosteric/endogenous 11-*cis*-retinal ligand [42,59]. The studies described above imply that rhodopsin contains different binding sites capable of accommodating several modulators at a time.

**Figure 3:**
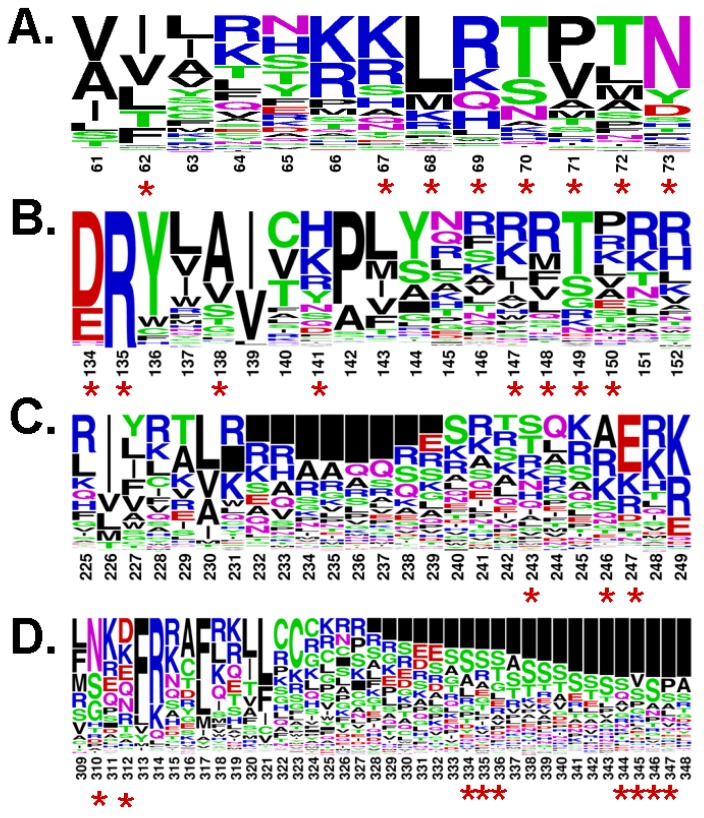
Frequency plot of the cytoplasmic region of Class A GPCRs. The frequency of residues corresponding to (A) CP loop 1, (B) CP loop 2, (C) CP loop 3, and (D) C-terminus of class A GPCRs. The amino acids at each position in the plot are colored based on their amino acid properties. A similar color between two amino acids at a particular position indicates that they both belong to the same group. The residues that were predicted bind to Ce6 on rhodopsin in the CP domain are indicated by red asterisks. The frequency plots were generated using the weblogo online tool [60].

A recent virtual ligand screening study to identify allosteric ligands that can stabilize GPCRs in different conformations lead to the identification of small molecule ligands that bind at the CP interface [45,46]. The compounds identified from an initial screen using the National Cancer Institute (NCI) compound library stabilized the Meta-II state and inhibited G-protein interactions, leading to the inhibition of the downstream signaling cascade [45]. While the compounds identified here inhibited G-protein activation, a set of tetrazole peptidomimetic compounds identified in a more recent screen using the Maybridge HitFinder library did not interfere with the interaction between the G-protein and rhodopsin. Rather, these ligands were found to bind rhodopsin allosterically and stabilize it in its activated state [46]. The compounds from both screens were predicted to bind at the CP interface and the binding site was shown to overlap with that of the G-protein and its peptide derivative in the peptide-bound opsin crystal structure [61].

The above studies [44,45,46,61] have demonstrated the presence of a novel ligand binding pocket in the CP domain of rhodopsin where secondary ligands can bind. The presence of the CP small molecule ligand binding pocket in GPCRs is further supported by the presence of high connectivity residues (indicative of the presence of a binding site) in the CP domain of rhodopsin by Illingsworth *et al.* [62]. In this approach, connectivity of a residue refers to the number of its neighboring contacts within a distance of 5.5Å. High connectivity residues were scored using the K-means algorithm to identify potential binding sites [62]. In addition to the main retinal binding pocket, a high-connectivity site in the CP domain was predicted when searching for local connectivity residues with 85% cutoff of the maximal energy value for the protein [62]. 

What could be the significance of the discovery of a CP binding pocket in GPCRs for drug discovery? Understanding the mechanism of selective modulation of downstream signaling cascades by allosteric molecules will be beneficial for developing potential drugs that are targeted towards these receptors. Due to the similarities in signaling mechanisms and presence of fewer downstream signaling proteins, the CP interface in different GPCRs is relatively conserved compared to the vast family of GPCR members and their diverse endogenous ligands (Figure 3). For example, it may be possible that ligands binding in the CP domain could be developed into universal modulators of GPCR activity. This could have potential for the medical and drug development communities for developing therapeutics for cancers where multiple GPCRs can substitute for each other’s functions [63,64,65,66].

## 4. Analogy between the Orthosteric Transmembrane Binding Pocket in Rhodopsin and the Allosteric Binding Pocket in Metabotropic Glutamate Receptors: Insight into the Molecular Mechanisms of Allosteric Regulation

As described in Section 1, multiple ligand binding pockets exist in GPCRs and the same pocket may bind an orthosteric ligand in one GPCR and an allosteric ligand in another GPCR. Comparison between the roles these pockets play for their receptors can teach us about the mechanisms of allosteric communication in GPCRs. Here, we will describe the analogy between the orthosteric TM binding pocket in rhodopsin which is equivalent to the allosteric binding pocket in metabotropic glutamate receptors (mGluRs). These receptors are highly diverged in sequence – rhodopsin is a Class A GPCR, while mGluRs are Class C GPCRs. Despite the divergence in sequence, there appears to be structural and functional conservation of this pocket. 

The endogenous ligand binding domain of Class C GPCRs is located in a long N-terminal EC domain, referred to as a “Venus flytrap” [67]. This ligand binding pocket location is unusual when compared to other GPCR family members where the small molecule ligand binding domain is typically located in the TM domain near its interface with the EC domain (Class A GPCRs) or in the EC loops and N-terminus close to the TM domain (Class B GPCRs). The venus flytrap domain is an independent folding unit and retains full ligand binding capabilities even when removed from the actual receptor, *i.e.* the sequence corresponding to the seven TM helices and interconnecting loops. Thus, for this class of receptors, it is particularly intriguing to understand how the signal generated at the venus fly trap like domain upon endogenous ligand binding is communicated to the CP side and how this process can be modulated by allosteric sites present in the TM domain. mGluRs are representative members of Class C GPCRs which bind glutamate, an amino acid that functions as the major excitatory neurotransmitter in the brain. Thus, mGluRs play modulatory roles in neuronal processes such as anxiety, learning, memory and perception of pain [68]. Because of these roles they are attractive drug targets for treatment of neuronal dysfunctions including seizures, epilepsy, Parkinson’s Disease and night blindness [69,70,71]. Allosteric mGluR ligands are promising drug targets because of their modulatory effects – enhancing or suppressing the response of mGluRs to glutamate. Small changes in the chemical structures of allosteric ligands were shown to switch their modulatory effects, allowing for fine tuning of the receptor responses. Allosteric modulation of mGluRs is very well demonstrated and many allosteric ligands have been identified including positive, negative and neutral modulators. However, despite these pharmacological successes, the mechanisms by which allosteric modulation occurs are only beginning to be investigated. 

**Figure 4:**
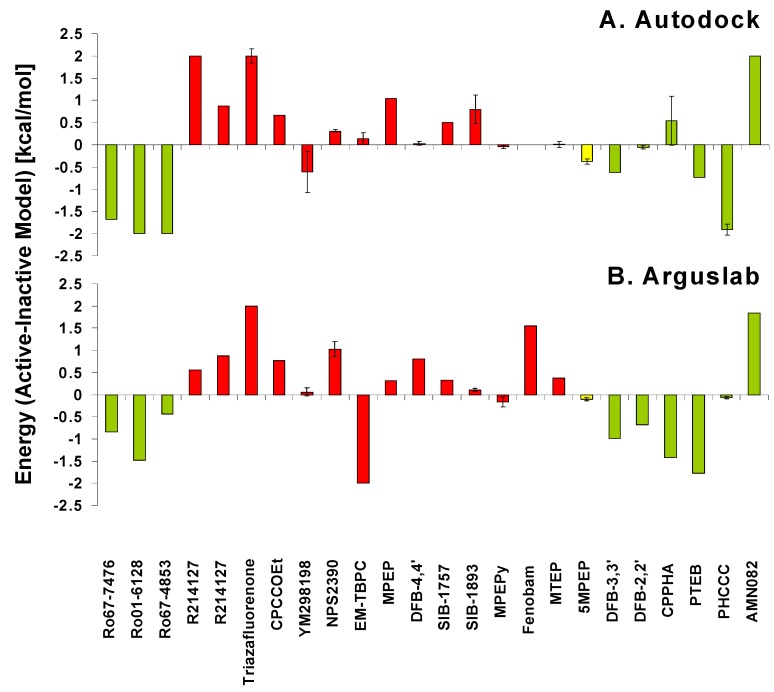
Differences in energy between active (Anisotropic Network Model based) and inactive (rhodopsin crystal-structure based) models of mGluRs. Green bars indicate positive modulators, red bars negative modulators and the yellow bar represents a neutral ligand. Where values of 2 are shown, the ligand did not dock to the active model, where values of −2 are shown, the ligand did not dock to the inactive model. Error bars indicate standard deviation in three docking experiments each for the respective active and inactive models. If an error bar is placed at a −2 or 2 bar, the error represents the standard deviation of the ligand and model combination where docking was observed. **A.** Results from docking with Autodock software. **B.** Results from docking with ArgusLab software. Image is adapted from [72].

A comparison between rhodopsin and mGluRs allows for a glimpse into the mechanism of action of allosteric modulators. With this aim, experimentally known allosteric mGluR ligands were docked to computationally generated models of different mGluR subtypes based on structures of rhodopsin [72]. Two different conformations of mGluRs were generated based on rhodopsin structures, namely one representing the inactive conformation, in rhodopsin, the 11-*cis* retinal bound dark state structure, and one representing the activated conformation. The results strongly supported the experimental finding that the allosteric ligand binding pockets of mGluRs are highly overlapping with the retinal binding pocket of rhodopsin. Furthermore, ligands showed clear preferences for the active and inactive states depending on their modulatory nature (Figure 4). In particular, the positive and negative modulators docked preferentially to the active and inactive models of the receptors, respectively. This finding suggests that the positive and negative mGluR modulators can be distinguished by their higher affinities for the active and inactive conformations of the receptors, respectively (Figure 4). Thus, mGluR allosteric modulation may occur via the stabilization of different conformations analogous to those identified in rhodopsin where they are induced by photochemical isomerization of the retinal ligand – despite the differences in sequences between mGluRs and rhodopsin. This versatility in accommodating allosteric and endogenous ligands for different members of the GPCR family suggests that the structural determinants for active and inactive functional states may be conserved across the GPCR family.

## 5. Pathways of Allosteric Communication

It is clear that every GPCR transmits the ligand binding signal occurring in the EC and/or TM domain to the CP domain via the TM domain because the CP domain is the site where the G protein recognizes the active conformation of the receptors. The presence of allosteric ligand binding sites in the TM and/or CP domain of GPCRs (Table 1) further supports that these sites might play a key role in transmitting the signal from the EC to CP side by modulating receptor structure at the different domains. Communication between the different ligand binding pockets in GPCRs will likely use the same structural mechanisms. It is thus expected that studies on understanding the molecular details of signal transmission/communication in the receptor from EC to CP domain can provide a better understanding of allosteric modulation and may thus provide the necessary insights for developing new therapeutic drugs that can selectively target subtype specific subtypes of GPCRs. Computational biology will likely take a key role in assisting with this task. A protein’s three-dimensional structure is determined by a complex network of interactions amongst the constituent residues and these interactions are often conserved within a family, *i.e.*, a set of proteins with highly similar structure and function. Therefore, the interactions can be modeled as a set of statistical constraints. These constraints can reflect position-specific conservations or correlated mutations. 

**Figure 5:**
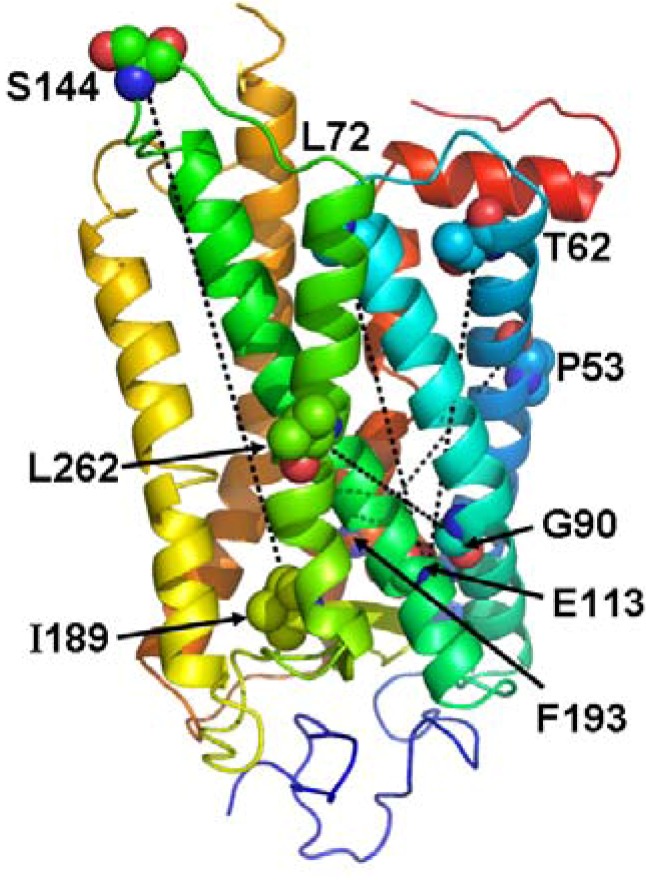
Rhodopsin structure highlighting the predicted network of communication path from the EC to CP domain. The significant edges representing the predicted network of interactions are highlighted as dashed lines and colored black. The residues that participate in the predicted network of interactions are labeled and rendered as spheres.

Ranganathan *et al*. have demonstrated that such constraints comprise virtually all the information required to specify a fold family [73,74,75]. Predicting the network of interactions in a protein structure or a protein family and to identify communication mechanisms of how distant sites in space communicate with each other is also referred to as the structure learning problem. As an input, a multiple sequence alignment (MSA) for a given family, can be used and the statistically significant constraints can be used to build a model [75,76,77]. Recently, Langmead and co-workers [78,79] have introduced an optimal algorithm for solving the structure learning problem. Their approach learns an undirected graphical model (also known as Markov Random Fields). This method was recently used to learn an undirected graphical model of the GPCR family [80]. In this method, an edge between a pair of residues is defined as the statistical coupling between two residue positions in the MSA. Along with predicting many edges between residues that are relatively close, the method also resulted in identifying very distant edges that are not close in the three-dimensional structure. The close edges are those that reside within one or few turns of a single helix, and across the helical bundle at a similar level. In contrast, distant edges are those that connect the beginnings and ends of helices or helix neighbors. The most significant subset of edges is shown mapped onto the structure of rhodopsin in Figure 5. The subsets of edges are between Glu113 to Leu72 and Thr62, Ile189 to Ser144, and Phe193 to Pro53 (Figure 5). Most of these edges connect residues near the endogenous (light-sensitive retinal) ligand binding pocket, including Glu113, the counter ion for the Schiff base, and thus part of the endogenous binding site, and residues in the CP domain. An edge is observed between Glu113 and Thr62, a highly conserved residue in the CP domain (Figure 3A) that has been implicated in receptor activation and G-protein specificity [81]. Further, this approach also predicted an edge between Glu113 and Leu72, another CP residue (Figure 3A). Leu72 is predicted to be part of the CP high-connectivity site [62]. It is also identified by PocketFinder analysis as a site of likely interactions (Figure 2B-C, red spheres). These findings support the hypothesis that there is a communication pathway between the endogenous and allosteric binding sites. This indicates that the communications predicted by this method are biologically meaningful. Thus, this example shows how it may be possible to learn from the resulting network structure, what are the underlying mechanisms of allosteric communication that ensure collaboration between the different ligand binding pockets. Once understood, these can be used for design of novel functionality.

## 6. Conclusions

The GPCR family is pharmacologically important because of the diverse ligands that target them and its role in numerous diseases. GPCRs have traditionally been inhibited or activated by antagonists and agonists. Recently, increased attention has been given to the understanding and development of drugs that bind at other sites, *i.e.* allosteric modulators. In this review, we surveyed the literature describing allosteric binding sites in GPCRs, including a novel site in the CP domain, and used rhodopsin as a model system to shed light on understanding the mechanisms of allosteric regulation in GPCRs. Specifically, we described general ligand binding pockets in all three domains (EC, TM and CP) that should in principal be available in all GPCRs. In some receptors, a particular pocket is orthosteric, while the same pocket becomes allosteric in others, depending on where the endogenous ligand binds. We show that the allosteric ligand binding pocket in Class C GPCRs is similar to the orthosteric TM binding pockets in Class A GPCRs. This implies a conservation of ligand binding pockets and the effects of ligand binding on conformational flexibility of the receptors. Studies on understanding the mechanisms of allosteric modulation may provide insights necessary for developing new therapeutic drugs that can selectively target subtype specific members of GPCRs.

## Abbreviations

GPCRs: G protein coupled receptors
TM: Transmembrane
EC: Extracellular
CP: Cytoplasmic
C3G: Cyanidin-3-glucoside
DFB-3-3’: 3,3’-difluorobenzaldazine
NHERF1: Na^+^/H^+^ exchange regulatory factor 1
PTH: Parathyroid hormone receptor
Ce6: Chlorin e6
mGluRs: Metabotropic glutamate receptors
ANM: Anisotropic Network Model
MSA: Multiple Sequence Alignment
